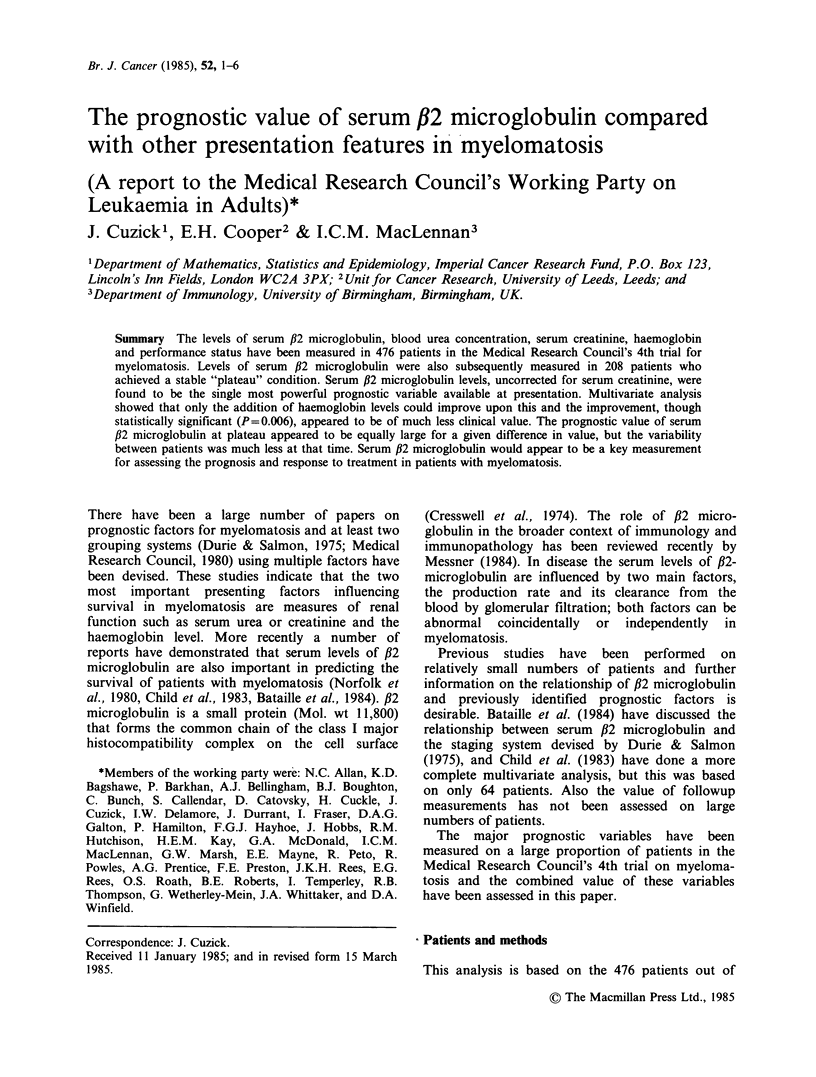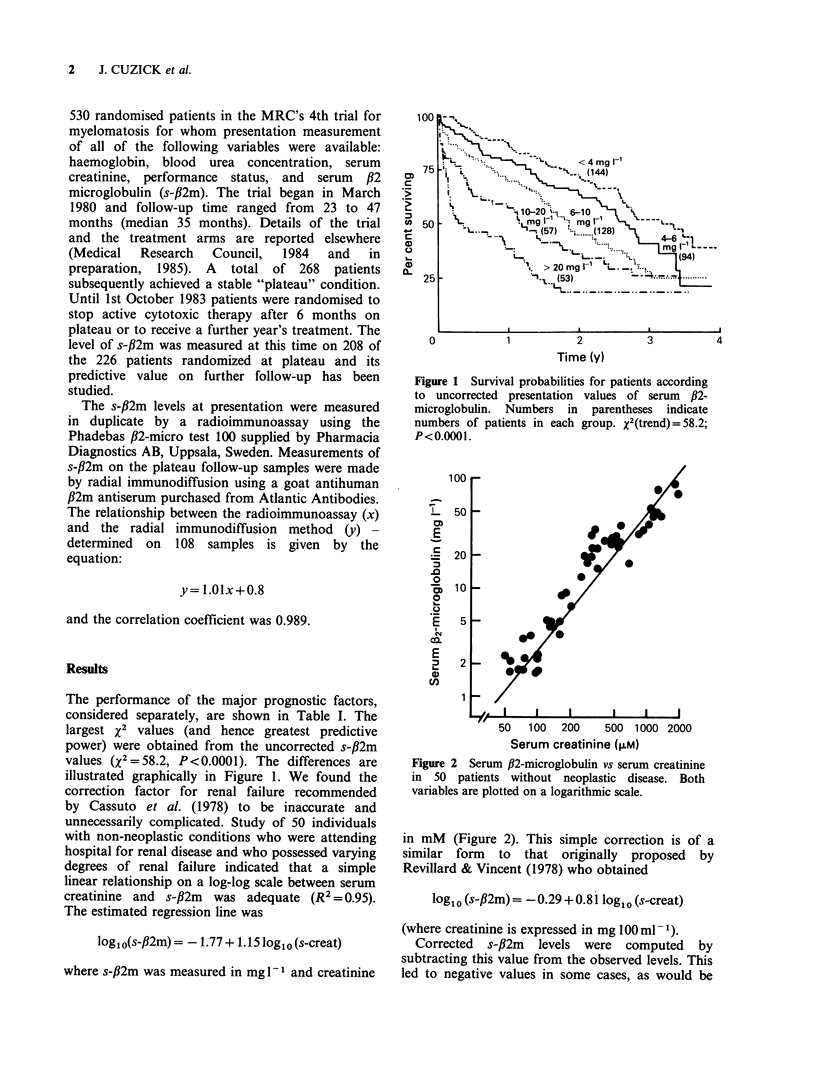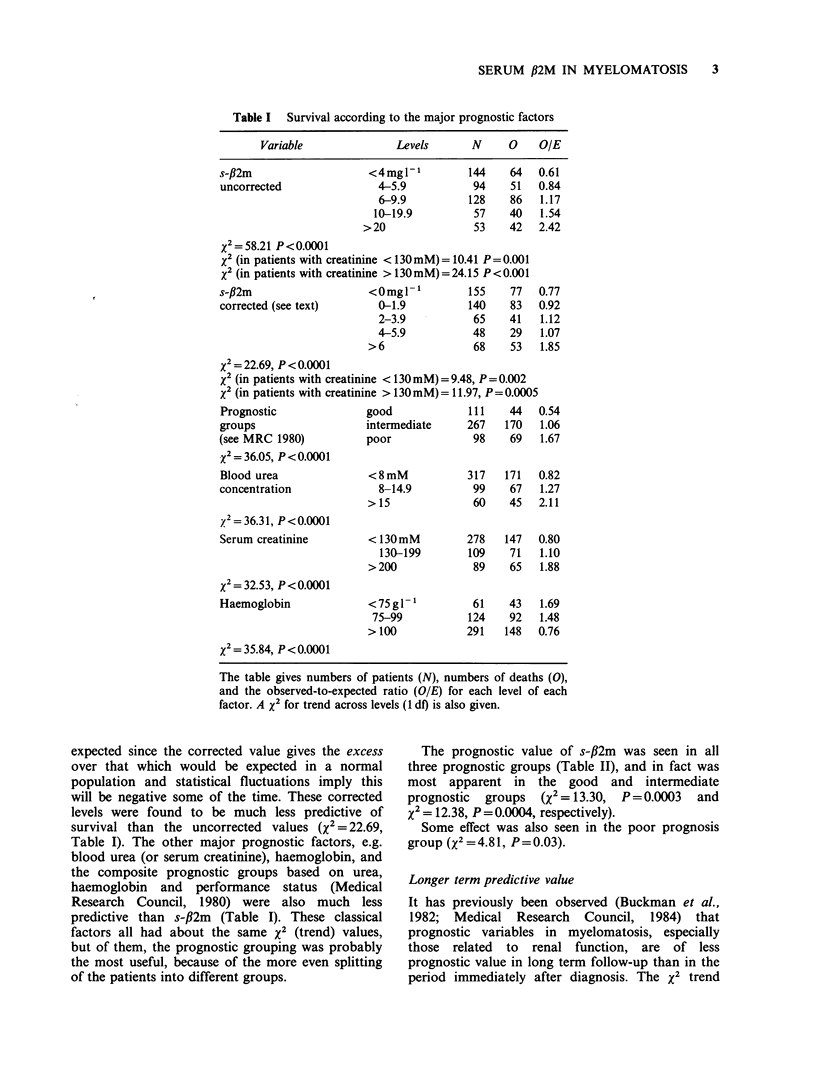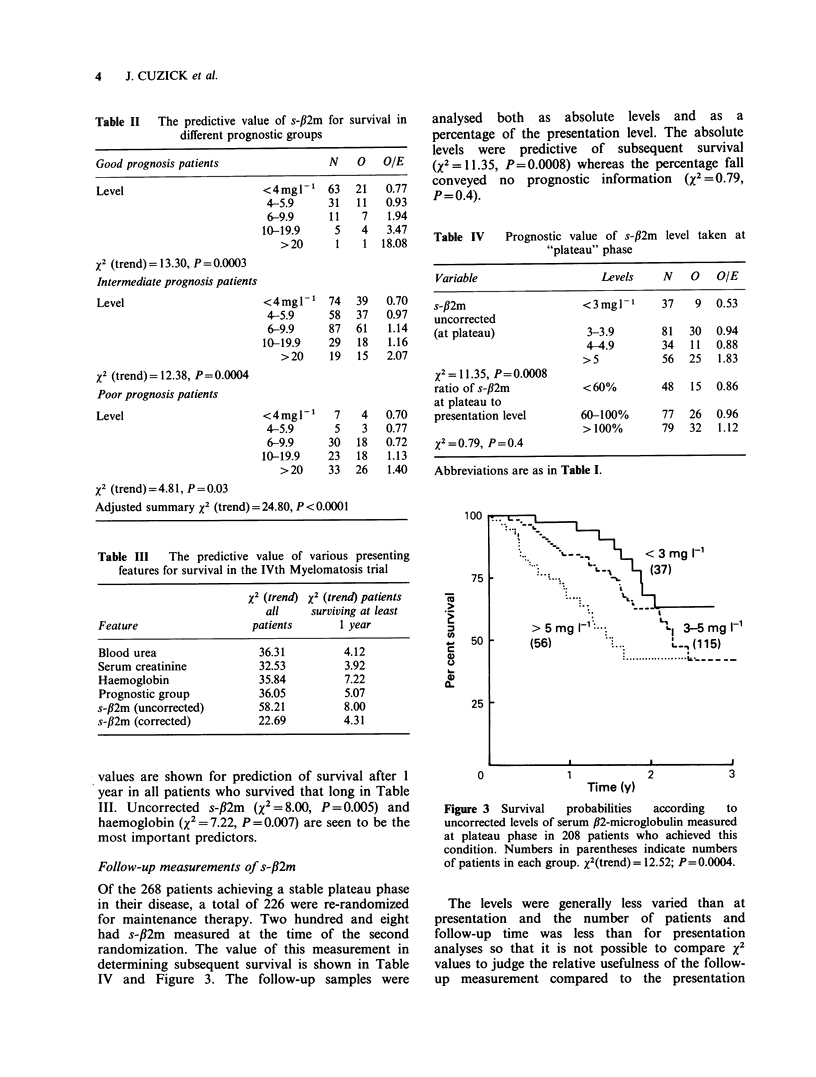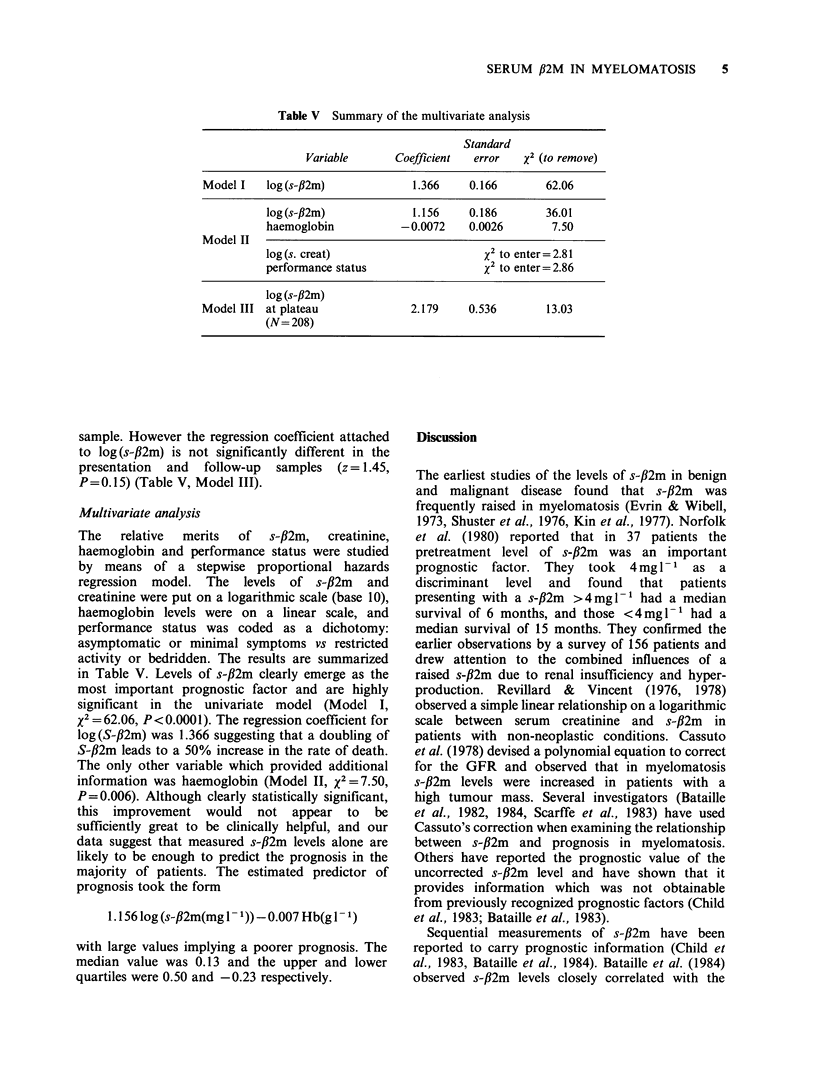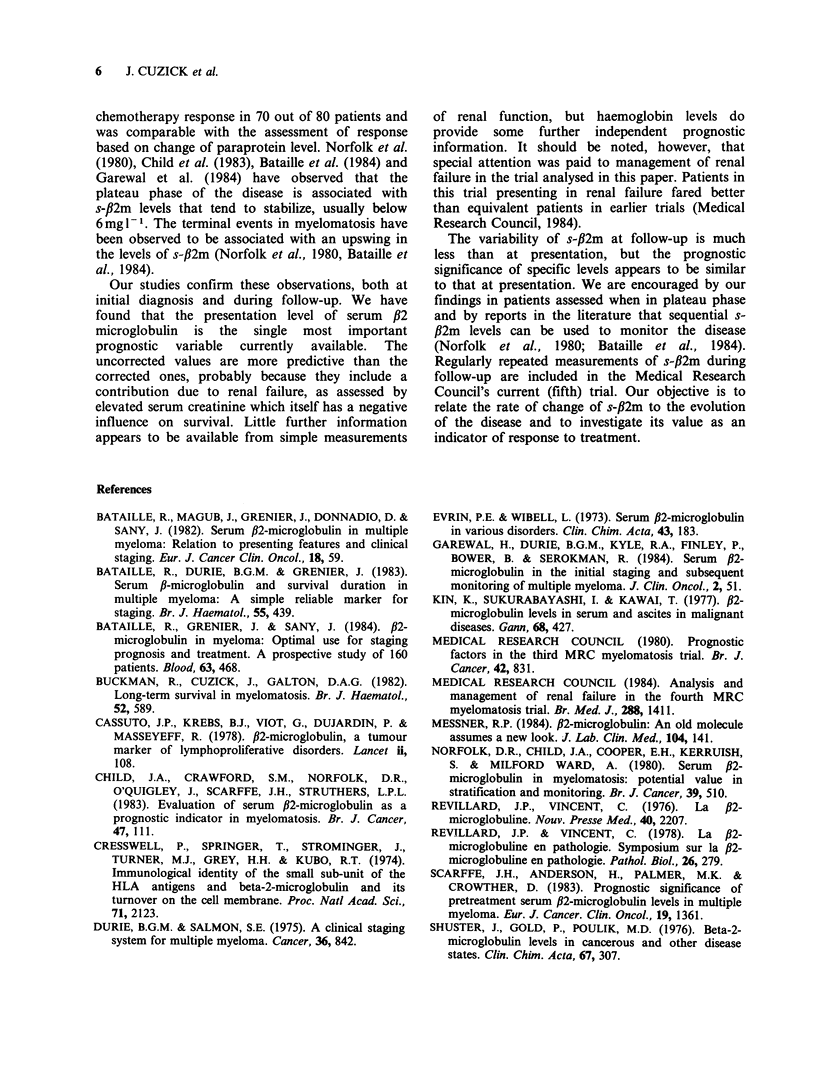# The prognostic value of serum beta 2 microglobulin compared with other presentation features in myelomatosis.

**DOI:** 10.1038/bjc.1985.140

**Published:** 1985-07

**Authors:** J. Cuzick, E. H. Cooper, I. C. MacLennan

## Abstract

The levels of serum beta 2 microglobulin, blood urea concentration, serum creatinine, haemoglobin and performance status have been measured in 476 patients in the Medical Research Council's 4th trial for myelomatosis. Levels of serum beta 2 microglobulin were also subsequently measured in 208 patients who achieved a stable "plateau" condition. Serum beta 2 microglobulin levels, uncorrected for serum creatinine, were found to be the single most powerful prognostic variable available at presentation. Multivariate analysis showed that only the addition of haemoglobin levels could improve upon this and the improvement, though statistically significant (P = 0.006), appeared to be of much less clinical value. The prognostic value of serum beta 2 microglobulin at plateau appeared to be equally large for a given difference in value, but the variability between patients was much less at that time. Serum beta 2 microglobulin would appear to be a key measurement for assessing the prognosis and response to treatment in patients with myelomatosis.


Br. J. Cancer (1985), 52, 1–6

### The prognostic value of serum β2 microglobulin compared with other presentation features in myelomatosis

### (A report to the Medical Research Council's Working Party on Leukaemia in Adults)*

J. Cuzick[1], E.H. Cooper[2] & I.C.M. MacLennan[3]

[1]*Department of Mathematics, Statistics and Epidemiology, Imperial Cancer Research Fund, P.O. Box 123, Lincoln's Inn Fields, London WC2A 3PX;* [2]*Unit for Cancer Research, University of Leeds, Leeds; and* [3]*Department of Immunology, University of Birmingham, Birmingham, UK.*

**Summary** The levels of serum β2 microglobulin, blood urea concentration, serum creatinine, haemoglobin and performance status have been measured in 476 patients in the Medical Research Council's 4th trial for myelomatosis. Levels of serum β2 microglobulin were also subsequently measured in 208 patients who achieved a stable "plateau" condition. Serum β2 microglobulin levels, uncorrected for serum creatinine, were found to be the single most powerful prognostic variable available at presentation. Multivariate analysis showed that only the addition of haemoglobin levels could improve upon this and the improvement, though statistically significant ($P = 0.006$), appeared to be of much less clinical value. The prognostic value of serum β2 microglobulin at plateau appeared to be equally large for a given difference in value, but the variability between patients was much less at that time. Serum β2 microglobulin would appear to be a key measurement for assessing the prognosis and response to treatment in patients with myelomatosis.

There have been a large number of papers on prognostic factors for myelomatosis and at least two grouping systems (Durie & Salmon, 1975; Medical Research Council, 1980) using multiple factors have been devised. These studies indicate that the two most important presenting factors influencing survival in myelomatosis are measures of renal function such as serum urea or creatinine and the haemoglobin level. More recently a number of reports have demonstrated that serum levels of β2 microglobulin are also important in predicting the survival of patients with myelomatosis (Norfolk *et al.*, 1980, Child *et al.*, 1983, Bataille *et al.*, 1984). β2 microglobulin is a small protein (Mol. wt 11,800) that forms the common chain of the class I major histocompatibility complex on the cell surface (Cresswell *et al.*, 1974). The role of β2 microglobulin in the broader context of immunology and immunopathology has been reviewed recently by Messner (1984). In disease the serum levels of β2-microglobulin are influenced by two main factors, the production rate and its clearance from the blood by glomerular filtration; both factors can be abnormal coincidentally or independently in myelomatosis.

Previous studies have been performed on relatively small numbers of patients and further information on the relationship of β2 microglobulin and previously identified prognostic factors is desirable. Bataille *et al.* (1984) have discussed the relationship between serum β2 microglobulin and the staging system devised by Durie & Salmon (1975), and Child *et al.* (1983) have done a more complete multivariate analysis, but this was based on only 64 patients. Also the value of followup measurements has not been assessed on large numbers of patients.

The major prognostic variables have been measured on a large proportion of patients in the Medical Research Council's 4th trial on myelomatosis and the combined value of these variables have been assessed in this paper.

### Patients and methods

This analysis is based on the 476 patients out of

*Members of the working party were: N.C. Allan, K.D. Bagshawe, P. Barkhan, A.J. Bellingham, B.J. Boughton, C. Bunch, S. Callendar, D. Catovsky, H. Cuckle, J. Cuzick, I.W. Delamore, J. Durrant, I. Fraser, D.A.G. Galton, P. Hamilton, F.G.J. Hayhoe, J. Hobbs, R.M. Hutchison, H.E.M. Kay, G.A. McDonald, I.C.M. MacLennan, G.W. Marsh, E.E. Mayne, R. Peto, R. Powles, A.G. Prentice, F.E. Preston, J.K.H. Rees, E.G. Rees, O.S. Roath, B.E. Roberts, I. Temperley, R.B. Thompson, G. Wetherley-Mein, J.A. Whittaker, and D.A. Winfield.

Correspondence: J. Cuzick.
Received 11 January 1985; and in revised form 15 March 1985.

© The Macmillan Press Ltd., 1985

530 randomised patients in the MRC's 4th trial for myelomatosis for whom presentation measurement of all of the following variables were available: haemoglobin, blood urea concentration, serum creatinine, performance status, and serum $\beta 2$ microglobulin ($s$-$\beta$2m). The trial began in March 1980 and follow-up time ranged from 23 to 47 months (median 35 months). Details of the trial and the treatment arms are reported elsewhere (Medical Research Council, 1984 and in preparation, 1985). A total of 268 patients subsequently achieved a stable "plateau" condition. Until 1st October 1983 patients were randomised to stop active cytotoxic therapy after 6 months on plateau or to receive a further year's treatment. The level of $s$-$\beta$2m was measured at this time on 208 of the 226 patients randomized at plateau and its predictive value on further follow-up has been studied.

The $s$-$\beta$2m levels at presentation were measured in duplicate by a radioimmunoassay using the Phadebas $\beta$2-micro test 100 supplied by Pharmacia Diagnostics AB, Uppsala, Sweden. Measurements of $s$-$\beta$2m on the plateau follow-up samples were made by radial immunodiffusion using a goat antihuman $\beta$2m antiserum purchased from Atlantic Antibodies. The relationship between the radioimmunoassay ($x$) and the radial immunodiffusion method ($y$) – determined on 108 samples is given by the equation:

$$y = 1.01x + 0.8$$

and the correlation coefficient was 0.989.

### Results

The performance of the major prognostic factors, considered separately, are shown in Table I. The largest $\chi^2$ values (and hence greatest predictive power) were obtained from the uncorrected $s$-$\beta$2m values ($\chi^2 = 58.2$, $P < 0.0001$). The differences are illustrated graphically in Figure 1. We found the correction factor for renal failure recommended by Cassuto *et al.* (1978) to be inaccurate and unnecessarily complicated. Study of 50 individuals with non-neoplastic conditions who were attending hospital for renal disease and who possessed varying degrees of renal failure indicated that a simple linear relationship on a log-log scale between serum creatinine and $s$-$\beta$2m was adequate ($R^2 = 0.95$). The estimated regression line was

$$\log_{10}(s\text{-}\beta 2\text{m}) = -1.77 + 1.15 \log_{10}(s\text{-creat})$$

where $s$-$\beta$2m was measured in mg $l^{-1}$ and creatinine

**Figure 1** Survival probabilities for patients according to uncorrected presentation values of serum $\beta$2-microglobulin. Numbers in parentheses indicate numbers of patients in each group. $\chi^2$(trend) = 58.2; $P < 0.0001$.

**Figure 2** Serum $\beta$2-microglobulin *vs* serum creatinine in 50 patients without neoplastic disease. Both variables are plotted on a logarithmic scale.

in mM (Figure 2). This simple correction is of a similar form to that originally proposed by Revillard & Vincent (1978) who obtained

$$\log_{10}(s\text{-}\beta 2\text{m}) = -0.29 + 0.81 \log_{10}(s\text{-creat})$$

(where creatinine is expressed in mg 100 ml$^{-1}$).

Corrected $s$-$\beta$2m levels were computed by subtracting this value from the observed levels. This led to negative values in some cases, as would be

**Table I** Survival according to the major prognostic factors

| *Variable* | *Levels* | *N* | *O* | *O/E* |
|---|---|---|---|---|
| $s$-$\beta$2m uncorrected | $<4\,mg\,l^{-1}$ | 144 | 64 | 0.61 |
| | 4–5.9 | 94 | 51 | 0.84 |
| | 6–9.9 | 128 | 86 | 1.17 |
| | 10–19.9 | 57 | 40 | 1.54 |
| | $>20$ | 53 | 42 | 2.42 |
| $\chi^2 = 58.21$ $P < 0.0001$ | | | | |
| $\chi^2$ (in patients with creatinine $<130\,mM) = 10.41$ $P = 0.001$ | | | | |
| $\chi^2$ (in patients with creatinine $>130\,mM) = 24.15$ $P < 0.001$ | | | | |
| $s$-$\beta$2m corrected (see text) | $<0\,mg\,l^{-1}$ | 155 | 77 | 0.77 |
| | 0–1.9 | 140 | 83 | 0.92 |
| | 2–3.9 | 65 | 41 | 1.12 |
| | 4–5.9 | 48 | 29 | 1.07 |
| | $>6$ | 68 | 53 | 1.85 |
| $\chi^2 = 22.69$, $P < 0.0001$ | | | | |
| $\chi^2$ (in patients with creatinine $<130\,mM) = 9.48$, $P = 0.002$ | | | | |
| $\chi^2$ (in patients with creatinine $>130\,mM) = 11.97$, $P = 0.0005$ | | | | |
| Prognostic groups (see MRC 1980) | good | 111 | 44 | 0.54 |
| | intermediate | 267 | 170 | 1.06 |
| | poor | 98 | 69 | 1.67 |
| $\chi^2 = 36.05$, $P < 0.0001$ | | | | |
| Blood urea concentration | $<8\,mM$ | 317 | 171 | 0.82 |
| | 8–14.9 | 99 | 67 | 1.27 |
| | $>15$ | 60 | 45 | 2.11 |
| $\chi^2 = 36.31$, $P < 0.0001$ | | | | |
| Serum creatinine | $<130\,mM$ | 278 | 147 | 0.80 |
| | 130–199 | 109 | 71 | 1.10 |
| | $>200$ | 89 | 65 | 1.88 |
| $\chi^2 = 32.53$, $P < 0.0001$ | | | | |
| Haemoglobin | $<75\,g\,l^{-1}$ | 61 | 43 | 1.69 |
| | 75–99 | 124 | 92 | 1.48 |
| | $>100$ | 291 | 148 | 0.76 |
| $\chi^2 = 35.84$, $P < 0.0001$ | | | | |

The table gives numbers of patients (*N*), numbers of deaths (*O*), and the observed-to-expected ratio (*O/E*) for each level of each factor. *A* $\chi^2$ for trend across levels (1 df) is also given.

expected since the corrected value gives the *excess* over that which would be expected in a normal population and statistical fluctuations imply this will be negative some of the time. These corrected levels were found to be much less predictive of survival than the uncorrected values ($\chi^2 = 22.69$, Table I). The other major prognostic factors, e.g. blood urea (or serum creatinine), haemoglobin, and the composite prognostic groups based on urea, haemoglobin and performance status (Medical Research Council, 1980) were also much less predictive than $s$-$\beta$2m (Table I). These classical factors all had about the same $\chi^2$ (trend) values, but of them, the prognostic grouping was probably the most useful, because of the more even splitting of the patients into different groups.

The prognostic value of $s$-$\beta$2m was seen in all three prognostic groups (Table II), and in fact was most apparent in the good and intermediate prognostic groups ($\chi^2 = 13.30$, $P = 0.0003$ and $\chi^2 = 12.38$, $P = 0.0004$, respectively).

Some effect was also seen in the poor prognosis group ($\chi^2 = 4.81$, $P = 0.03$).

### *Longer term predictive value*

It has previously been observed (Buckman *et al.*, 1982; Medical Research Council, 1984) that prognostic variables in myelomatosis, especially those related to renal function, are of less prognostic value in long term follow-up than in the period immediately after diagnosis. The $\chi^2$ trend

**Table II** The predictive value of *s*-$\beta$2m for survival in different prognostic groups

| *Good prognosis patients* | | *N* | *O* | *O/E* |
|---|---|---|---|---|
| Level | $<4\,mg\,l^{-1}$ | 63 | 21 | 0.77 |
| | 4–5.9 | 31 | 11 | 0.93 |
| | 6–9.9 | 11 | 7 | 1.94 |
| | 10–19.9 | 5 | 4 | 3.47 |
| | >20 | 1 | 1 | 18.08 |
| $\chi^2$ (trend) = 13.30, $P=0.0003$ | | | | |
| *Intermediate prognosis patients* | | | | |
| Level | $<4\,mg\,l^{-1}$ | 74 | 39 | 0.70 |
| | 4–5.9 | 58 | 37 | 0.97 |
| | 6–9.9 | 87 | 61 | 1.14 |
| | 10–19.9 | 29 | 18 | 1.16 |
| | >20 | 19 | 15 | 2.07 |
| $\chi^2$ (trend) = 12.38, $P=0.0004$ | | | | |
| *Poor prognosis patients* | | | | |
| Level | $<4\,mg\,l^{-1}$ | 7 | 4 | 0.70 |
| | 4–5.9 | 5 | 3 | 0.77 |
| | 6–9.9 | 30 | 18 | 0.72 |
| | 10–19.9 | 23 | 18 | 1.13 |
| | >20 | 33 | 26 | 1.40 |
| $\chi^2$ (trend) = 4.81, $P=0.03$ | | | | |
| Adjusted summary $\chi^2$ (trend) = 24.80, $P<0.0001$ | | | | |

**Table III** The predictive value of various presenting features for survival in the IVth Myelomatosis trial

| *Feature* | $\chi^2$ *(trend) all patients* | $\chi^2$ *(trend) patients surviving at least 1 year* |
|---|---|---|
| Blood urea | 36.31 | 4.12 |
| Serum creatinine | 32.53 | 3.92 |
| Haemoglobin | 35.84 | 7.22 |
| Prognostic group | 36.05 | 5.07 |
| *s*-$\beta$2m (uncorrected) | 58.21 | 8.00 |
| *s*-$\beta$2m (corrected) | 22.69 | 4.31 |

values are shown for prediction of survival after 1 year in all patients who survived that long in Table III. Uncorrected *s*-$\beta$2m ($\chi^2=8.00$, $P=0.005$) and haemoglobin ($\chi^2=7.22$, $P=0.007$) are seen to be the most important predictors.

### *Follow-up measurements of s-$\beta$2m*

Of the 268 patients achieving a stable plateau phase in their disease, a total of 226 were re-randomized for maintenance therapy. Two hundred and eight had *s*-$\beta$2m measured at the time of the second randomization. The value of this measurement in determining subsequent survival is shown in Table IV and Figure 3. The follow-up samples were analysed both as absolute levels and as a percentage of the presentation level. The absolute levels were predictive of subsequent survival ($\chi^2=11.35$, $P=0.0008$) whereas the percentage fall conveyed no prognostic information ($\chi^2=0.79$, $P=0.4$).

**Table IV** Prognostic value of *s*-$\beta$2m level taken at "plateau" phase

| *Variable* | *Levels* | *N* | *O* | *O/E* |
|---|---|---|---|---|
| *s*-$\beta$2m uncorrected | $<3\,mg\,l^{-1}$ | 37 | 9 | 0.53 |
| (at plateau) | 3–3.9 | 81 | 30 | 0.94 |
| | 4–4.9 | 34 | 11 | 0.88 |
| | >5 | 56 | 25 | 1.83 |
| $\chi^2=11.35$, $P=0.0008$ | | | | |
| ratio of *s*-$\beta$2m | <60% | 48 | 15 | 0.86 |
| at plateau to presentation level | 60–100% | 77 | 26 | 0.96 |
| | >100% | 79 | 32 | 1.12 |
| $\chi^2=0.79$, $P=0.4$ | | | | |

Abbreviations are as in **Table I**.

**Figure 3** Survival probabilities according to uncorrected levels of serum $\beta$2-microglobulin measured at plateau phase in 208 patients who achieved this condition. Numbers in parentheses indicate numbers of patients in each group. $\chi^2$(trend) = 12.52; $P=0.0004$.

The levels were generally less varied than at presentation and the number of patients and follow-up time was less than for presentation analyses so that it is not possible to compare $\chi^2$ values to judge the relative usefulness of the follow-up measurement compared to the presentation

**Table V** Summary of the multivariate analysis

| | *Variable* | *Coefficient* | *Standard error* | $\chi^2$ (*to remove*) |
|---|---|---|---|---|
| Model I | log ($s$-$\beta$2m) | 1.366 | 0.166 | 62.06 |
| Model II | log ($s$-$\beta$2m) | 1.156 | 0.186 | 36.01 |
| | haemoglobin | −0.0072 | 0.0026 | 7.50 |
| | log ($s$. creat) | | $\chi^2$ to enter = 2.81 | |
| | performance status | | $\chi^2$ to enter = 2.86 | |
| Model III | log ($s$-$\beta$2m) at plateau ($N$ = 208) | 2.179 | 0.536 | 13.03 |

sample. However the regression coefficient attached to log ($s$-$\beta$2m) is not significantly different in the presentation and follow-up samples ($z = 1.45$, $P = 0.15$) (Table V, Model III).

*Multivariate analysis*

The relative merits of $s$-$\beta$2m, creatinine, haemoglobin and performance status were studied by means of a stepwise proportional hazards regression model. The levels of $s$-$\beta$2m and creatinine were put on a logarithmic scale (base 10), haemoglobin levels were on a linear scale, and performance status was coded as a dichotomy: asymptomatic or minimal symptoms *vs* restricted activity or bedridden. The results are summarized in Table V. Levels of $s$-$\beta$2m clearly emerge as the most important prognostic factor and are highly significant in the univariate model (Model I, $\chi^2 = 62.06$, $P < 0.0001$). The regression coefficient for log ($S$-$\beta$2m) was 1.366 suggesting that a doubling of $S$-$\beta$2m leads to a 50% increase in the rate of death. The only other variable which provided additional information was haemoglobin (Model II, $\chi^2 = 7.50$, $P = 0.006$). Although clearly statistically significant, this improvement would not appear to be sufficiently great to be clinically helpful, and our data suggest that measured $s$-$\beta$2m levels alone are likely to be enough to predict the prognosis in the majority of patients. The estimated predictor of prognosis took the form

$$1.156 \log (s\text{-}\beta 2\text{m}(\text{mg l}^{-1})) - 0.007\,\text{Hb}(\text{g l}^{-1})$$

with large values implying a poorer prognosis. The median value was 0.13 and the upper and lower quartiles were 0.50 and −0.23 respectively.

### Discussion

The earliest studies of the levels of $s$-$\beta$2m in benign and malignant disease found that $s$-$\beta$2m was frequently raised in myelomatosis (Evrin & Wibell, 1973, Shuster *et al.*, 1976, Kin *et al.*, 1977). Norfolk *et al.* (1980) reported that in 37 patients the pretreatment level of $s$-$\beta$2m was an important prognostic factor. They took 4 mg l$^{-1}$ as a discriminant level and found that patients presenting with a $s$-$\beta$2m > 4 mg l$^{-1}$ had a median survival of 6 months, and those < 4 mg l$^{-1}$ had a median survival of 15 months. They confirmed the earlier observations by a survey of 156 patients and drew attention to the combined influences of a raised $s$-$\beta$2m due to renal insufficiency and hyper-production. Revillard & Vincent (1976, 1978) observed a simple linear relationship on a logarithmic scale between serum creatinine and $s$-$\beta$2m in patients with non-neoplastic conditions. Cassuto *et al.* (1978) devised a polynomial equation to correct for the GFR and observed that in myelomatosis $s$-$\beta$2m levels were increased in patients with a high tumour mass. Several investigators (Bataille *et al.*, 1982, 1984, Scarffe *et al.*, 1983) have used Cassuto's correction when examining the relationship between $s$-$\beta$2m and prognosis in myelomatosis. Others have reported the prognostic value of the uncorrected $s$-$\beta$2m level and have shown that it provides information which was not obtainable from previously recognized prognostic factors (Child *et al.*, 1983; Bataille *et al.*, 1983).

Sequential measurements of $s$-$\beta$2m have been reported to carry prognostic information (Child *et al.*, 1983, Bataille *et al.*, 1984). Bataille *et al.* (1984) observed $s$-$\beta$2m levels closely correlated with the chemotherapy response in 70 out of 80 patients and was comparable with the assessment of response based on change of paraprotein level. Norfolk *et al.* (1980), Child *et al.* (1983), Bataille *et al.* (1984) and Garewal et al. (1984) have observed that the plateau phase of the disease is associated with *s*-$\beta$2m levels that tend to stabilize, usually below $6\,mg\,l^{-1}$. The terminal events in myelomatosis have been observed to be associated with an upswing in the levels of *s*-$\beta$2m (Norfolk *et al.*, 1980, Bataille *et al.*, 1984).

Our studies confirm these observations, both at initial diagnosis and during follow-up. We have found that the presentation level of serum $\beta$2 microglobulin is the single most important prognostic variable currently available. The uncorrected values are more predictive than the corrected ones, probably because they include a contribution due to renal failure, as assessed by elevated serum creatinine which itself has a negative influence on survival. Little further information appears to be available from simple measurements of renal function, but haemoglobin levels do provide some further independent prognostic information. It should be noted, however, that special attention was paid to management of renal failure in the trial analysed in this paper. Patients in this trial presenting in renal failure fared better than equivalent patients in earlier trials (Medical Research Council, 1984).

The variability of *s*-$\beta$2m at follow-up is much less than at presentation, but the prognostic significance of specific levels appears to be similar to that at presentation. We are encouraged by our findings in patients assessed when in plateau phase and by reports in the literature that sequential *s*-$\beta$2m levels can be used to monitor the disease (Norfolk *et al.*, 1980; Bataille *et al.*, 1984). Regularly repeated measurements of *s*-$\beta$2m during follow-up are included in the Medical Research Council's current (fifth) trial. Our objective is to relate the rate of change of *s*-$\beta$2m to the evolution of the disease and to investigate its value as an indicator of response to treatment.

### References

BATAILLE, R., MAGUB, J., GRENIER, J., DONNADIO, D. & SANY, J. (1982). Serum $\beta$2-microglobulin in multiple myeloma: Relation to presenting features and clinical staging. *Eur. J. Cancer Clin. Oncol.*, **18,** 59.

BATAILLE, R., DURIE, B.G.M. & GRENIER, J. (1983). Serum $\beta$-microglobulin and survival duration in multiple myeloma: A simple reliable marker for staging. *Br. J. Haematol.*, **55,** 439.

BATAILLE, R., GRENIER, J. & SANY, J. (1984). $\beta$2-microglobulin in myeloma: Optimal use for staging prognosis and treatment. A prospective study of 160 patients. *Blood*, **63,** 468.

BUCKMAN, R., CUZICK, J., GALTON, D.A.G. (1982). Long-term survival in myelomatosis. *Br. J. Haematol.*, **52,** 589.

CASSUTO, J.P., KREBS, B.J., VIOT, G., DUJARDIN, P. & MASSEYEFF, R. (1978). $\beta$2-microglobulin, a tumour marker of lymphoproliferative disorders. *Lancet* **ii,** 108.

CHILD, J.A., CRAWFORD, S.M., NORFOLK, D.R., O'QUIGLEY, J., SCARFFE, J.H., STRUTHERS, L.P.L. (1983). Evaluation of serum $\beta$2-microglobulin as a prognostic indicator in myelomatosis. *Br. J. Cancer*, **47,** 111.

CRESSWELL, P., SPRINGER, T., STROMINGER, J., TURNER, M.J., GREY, H.H. & KUBO, R.T. (1974). Immunological identity of the small sub-unit of the HLA antigens and beta-2-microglobulin and its turnover on the cell membrane. *Proc. Natl Acad. Sci.*, **71,** 2123.

DURIE, B.G.M. & SALMON, S.E. (1975). A clinical staging system for multiple myeloma. *Cancer*, **36,** 842.

EVRIN, P.E. & WIBELL, L. (1973). Serum $\beta$2-microglobulin in various disorders. *Clin. Chim. Acta*, **43,** 183.

GAREWAL, H., DURIE, B.G.M., KYLE, R.A., FINLEY, P., BOWER, B. & SEROKMAN, R. (1984). Serum $\beta$2-microglobulin in the initial staging and subsequent monitoring of multiple myeloma. *J. Clin. Oncol.*, **2,** 51.

KIN, K., SUKURABAYASHI, I. & KAWAI, T. (1977). $\beta$2-microglobulin levels in serum and ascites in malignant diseases. *Gann*, **68,** 427.

MEDICAL RESEARCH COUNCIL (1980). Prognostic factors in the third MRC myelomatosis trial. *Br. J. Cancer*, **42,** 831.

MEDICAL RESEARCH COUNCIL (1984). Analysis and management of renal failure in the fourth MRC myelomatosis trial. *Br. Med. J.*, **288,** 1411.

MESSNER, R.P. (1984). $\beta$2-microglobulin: An old molecule assumes a new look. *J. Lab. Clin. Med.*, **104,** 141.

NORFOLK, D.R., CHILD, J.A., COOPER, E.H., KERRUISH, S. & MILFORD WARD, A. (1980). Serum $\beta$2-microglobulin in myelomatosis: potential value in stratification and monitoring. *Br. J. Cancer*, **39,** 510.

REVILLARD, J.P., VINCENT, C. (1976). La $\beta$2-microglobuline. *Nouv. Presse Med.*, **40,** 2207.

REVILLARD, J.P. & VINCENT, C. (1978). La $\beta$2-microglobuline en pathologie. Symposium sur la $\beta$2-microglobuline en pathologie. *Pathol. Biol.*, **26,** 279.

SCARFFE, J.H., ANDERSON, H., PALMER, M.K. & CROWTHER, D. (1983). Prognostic significance of pretreatment serum $\beta$2-microglobulin levels in multiple myeloma. *Eur. J. Cancer. Clin. Oncol.*, **19,** 1361.

SHUSTER, J., GOLD, P., POULIK, M.D. (1976). Beta-2-microglobulin levels in cancerous and other disease states. *Clin. Chim. Acta*, **67,** 307.